# Herlyn-Werner-Wunderlich syndrome in three infants: a case report of surgical treatment

**DOI:** 10.3389/fped.2026.1912583

**Published:** 2026-08-12

**Authors:** Jiarong Chen, Youcai Feng, Jiabo Chen, Danping Zeng, Hua Li, Hongjun Gao

**Affiliations:** 1 Graduate School, Guangxi University of Chinese Medicine, Nanning, China; 2 Department of Urology, Guangxi Zhuang Autonomous Region Maternal and Child Health Hospital, Nanning, China; 3 Department of Urology, The Second Affiliated Hospital of Guangxi University of Chinese Medicine, Nanning, China

**Keywords:** congenital reproductive tract malformation, Herlyn-Werner-Wunderlich syndrome, infant, laparoscopic assistance, surgical treatmen, transvaginal incision and fistulization

## Abstract

Oblique vaginal septum syndrome (Herlyn-Werner-Wunderlich syndrome) is a congenital reproductive tract malformation frequently accompanied by ipsilateral renal dysplasia. During infancy, it can lead to urinary tract infections, dysuria, and abdominal masses. Delayed intervention predisposes patients to hydronephrosis and even renal failure. Current treatment primarily involves incision and drainage; however, traditional transvaginal surgery in infants is limited by a restricted surgical field and a lack of standardized protocols. This case report presents the clinical data of three affected infants (age range: 5 days to 4 months and 15 days) treated at our institution, evaluating the efficacy of two minimally invasive surgical approaches. During a follow-up period of 1–12 months postoperatively, all patients achieved complete recovery of normal voiding function, with no recurrence of urinary tract infections, and no instances of intestinal injury, hemorrhage, or restenosis were observed. Transvaginal direct incision is considered suitable for patients presenting with vulvar masses caused by a low-positioned oblique septum, whereas a laparoscopy-assisted approach effectively optimizes surgical conditions for cases involving a high-positioned oblique septum or those with significant inflammation following repeated punctures. Both techniques resulted in symptom relief without major complications. We emphasize the importance of early and precise intervention, as well as the avoidance of blind puncture, to preserve renal function.

## 1 Introduction

Herlyn-Werner-Wunderlich syndrome, also referred to as obstructed hemivagina with ipsilateral renal agenesis (OHVIRA) syndrome, is a rare congenital anomaly of the female reproductive tract. It is characterized by the triad of didelphys uterus, double cervix, unilateral obstructed hemivagina, and ipsilateral renal agenesis (1). The embryological basis of this condition involves a concurrent developmental disorder of the paramesonephric (Müllerian) and mesonephric (Wolffian) ducts, leading to structural abnormalities in both the urinary and reproductive systems. In infancy, this malformation can present with significant symptoms. The accumulation of secretions or urine in the obstructed retrovaginal space can rapidly form a vaginal or pelvic mass, subsequently causing voiding difficulties, recurrent urinary tract infections, and potentially obstructive renal failure (2).

Given the potential for severe complications, early diagnosis and timely intervention for Herlyn-Werner-Wunderlich syndrome are critical issues in the fields of urogynecology and pediatric surgery. Currently, pelvic ultrasonography and magnetic resonance imaging (MRI) are the primary diagnostic modalities. Ultrasonography can effectively screen for urogenital anomalies, while MRI precisely delineates the uterine morphology, the location of the obstruction, and the complex anatomical relationships with surrounding organs, providing essential information for surgical planning (3). The core treatment principle is the timely relief of obstruction, drainage of accumulated pus or fluid, and restoration of genital tract patency. The current mainstream therapeutic strategy is transvaginal or hysteroscopic septal incision and fenestration. This minimally invasive procedure has become the preferred treatment for adolescent and adult patients (4). However, the vast majority of existing clinical studies and treatment guidelines focus on the classic patient population of post-menarcheal adolescents and adults presenting with dysmenorrhea and pelvic masses due to hematocolpos (5). A significant research gap and technical bottleneck exist in the diagnosis and treatment of Herlyn-Werner-Wunderlich syndrome during infancy. Most available literature is limited to case reports, which predominantly describe a single approach of transvaginal puncture or incision (6). This case report presents three infant cases of oblique vaginal septum syndrome with distinct clinical manifestations, with the aim of exploring and analyzing the applicability of two minimally invasive surgical strategies: transvaginal direct septal incision and ostomy for low-level obstruction, and laparoscopic-assisted transvaginal septal incision and ostomy for high-level or complex obstruction.

## 2 Case report

### 2.1 General information

This case report collected clinical data from the Maternal and Child Health Hospital of Guangxi Zhuang Autonomous Region three infants with Herlyn-Werner-Wunderlich syndrome (HWWS) who underwent surgical treatment between May 2024 and May 2026. The study involving human participants was approved by the Medical Ethics Committee of the Maternal and Child Health Hospital of Guangxi Zhuang Autonomous Region. The research was conducted in accordance with local regulations and institutional requirements. Written informed consent for participation in this study was obtained from the legal guardians/next of kin of the participants. For any potentially identifiable images or data included in this article, informed consent was obtained from the relevant individuals and the legal guardians/next of kin of the minors.

#### 2.1.1 Case 1

**Figure 1:**
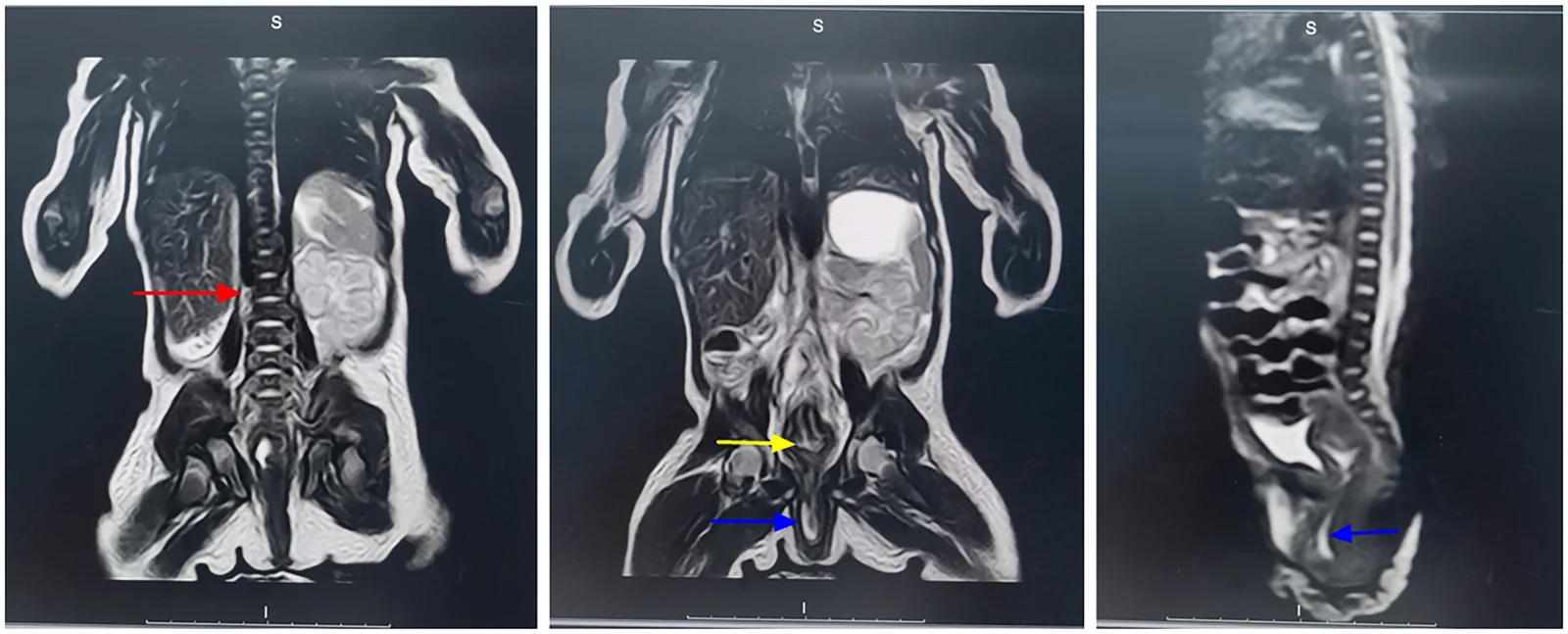
Magnetic resonance imaging (MRI) findings: right hypoplastic kidney (red arrow) **(A)**; uterine malformation (bilateral uterine cavities and cervixes) (yellow arrow) **(B)**; right vaginal fluid accumulation suggestive of vaginal septum oblique formation (blue arrow) **(C)**.

A 5-day-old female infant was hospitalized with a vulvar mass detected 2 days prior. Urination was associated with urine bifurcation and crying. Physical examination revealed a normal female external genital structure with a cystic mass measuring12mm × 10mm × 10 mm in the vulvar region. The urethral orifice, located superior to the mass, was morphologically normal. Laboratory tests showed a urinary leukocyte count of 116 cells/*μ*L. Ultrasonography identified a right pelvic ectopic kidney with hydronephrosis and a hypoechoic area posterior to the bladder, suggestive of a ureteral origin. MRI Findings: The MRI revealed possible dysplastic renal tissue on the right side, uterine malformation (dual uterine cavities and dual cervices), and fluid accumulation in the right vagina, suggesting the formation of an oblique vaginal septum, indicative of Herlyn-Werner-Wunderlich syndrome (Figure 1). During hospitalization, incision and marsupialization of the oblique vaginal septum were performed under direct visualization.

#### 2.1.2 Case 2

**Figure 2:**
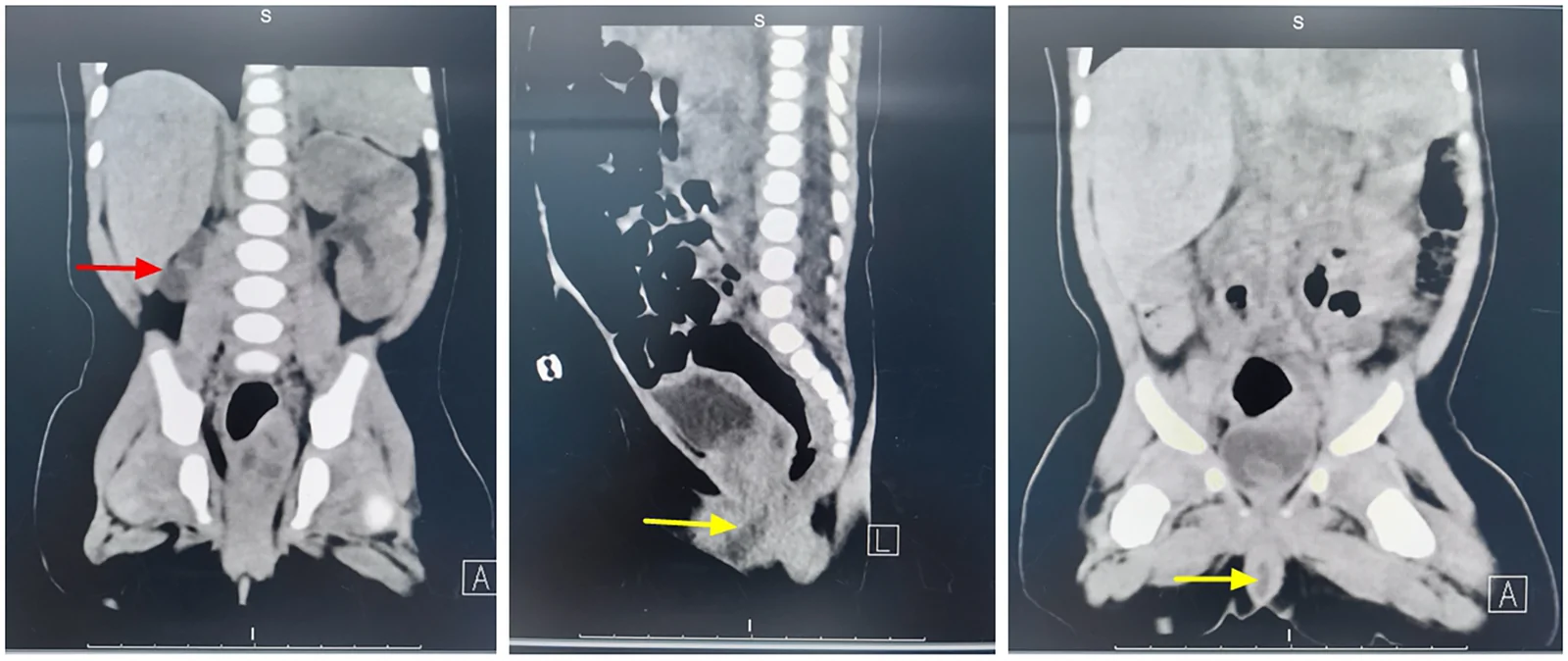
CT findings: Right side of the kidney with hypoplasia and polycystic changes (red arrow) **(A)**; the lower segment of the uterus and cervix appear enlarged; fluid accumulation in the uterine cavity and vagina (yellow arrows) **(B,C)**.

A 1-month-4-day-old female infant was admitted following a 1-month history of a vulvar mass. Urine bifurcation was observed during urination without crying. Physical examination demonstrated a normal female external genital appearance with a cystic mass (10 mm × 10 mm × 10 mm) in the vulvar region. The urethral orifice, located above the mass, appeared normal. Laboratory findings included a urinary leukocyte count of 56 cells/μL, a urine culture positive for Escherichia coli, and an alpha-fetoprotein (AFP) level of 2013.00 ng/mL. Ultrasonography showed multicystic dysplastic kidney (MCDK) of the right kidney, a ureterocele within the bladder, and no significant abnormalities in the left kidney. CT Findings: The CT scan showed a dysplastic right kidney with polycystic changes, a right ureterocele within the bladder, a full appearance of the lower uterine segment and cervix, and fluid accumulation in the uterine cavity and vagina, indicative of Herlyn-Werner-Wunderlich syndrome (Figure 2). During hospitalization, incision and marsupialization of the vaginal septum were performed under direct visualization.

#### 2.1.3 Case 3

**Figure 3:**
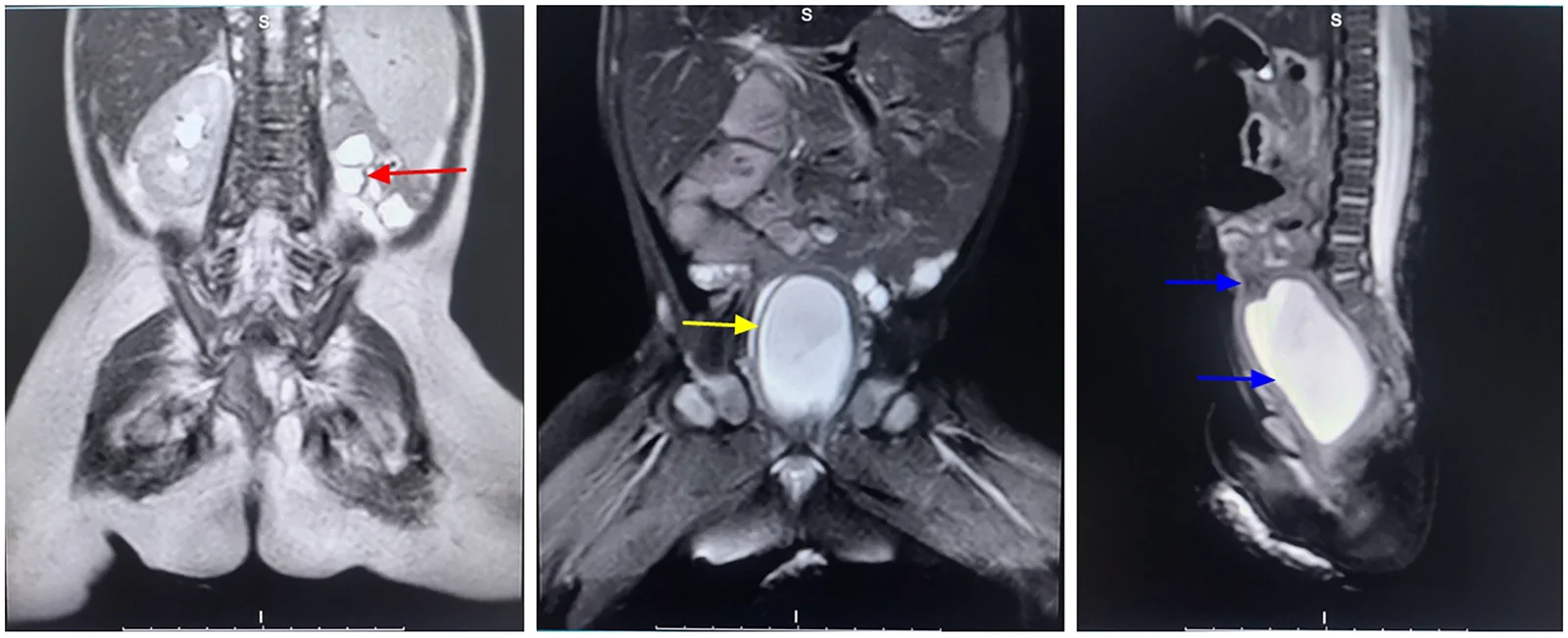
MRI findings: Left-sided polycystic kidney with dysplasia (red arrow) **(A)**; formation of a left vaginal septum with associated vaginal effusion (yellow arrow) **(B)**; bilateral cervical abnormalities and significant vaginal fluid accumulation (blue arrow) **(C)**.

A 4-month-15-day-old female infant was hospitalized due to a recurrent abdominal mass over 4 months. The patient's prior magnetic resonance imaging findings included: left multicystic dysplastic kidney (red arrow), left oblique vaginal septum with hydrocolpos (yellow arrow), double cervix and massive hydrocolpos (blue arrow), suggestive of oblique vaginal septum syndrome (Figure 3). Under ultrasound guidance, transabdominal puncture and aspiration yielded 75 mL of pale yellow, clear fluid. Subsequently, monthly ultrasound examinations consistently revealed significant hydrocolpos, and the patient underwent three sessions of scheduled puncture and aspiration, with 60–80 mL of fluid withdrawn each time. The patient was admitted to the hospital due to “purulent vaginal discharge.” On physical examination, the external genitalia appeared normal, with a normal urethral orifice and vaginal introitus. A small amount of malodorous purulent discharge was observed at the vaginal orifice. Laboratory findings: Enterococcus faecium was cultured from both urine and purulent discharge. CA19-9: 94.80 U/mL; AFP: 51,087.00 ng/mL. Ultrasound examination revealed a fluid collection in the vagina measuring 52 mm × 27 mm × 34 mm, with poor internal echogenicity, scattered linear echoes, and flocculent echogenic deposits. The left kidney was multicystic and dysplastic; the right kidney was compensatory enlarged with hydronephrosis. Bilateral ureters were diffusely tortuous and dilated. During hospitalization, laparoscopic-assisted vaginal septal incision and marsupialization were performed, and a drainage tube was placed. Postoperatively, 50–70 mL of fluid was drained daily. The drainage tube was removed on postoperative day 5, and the patient was subsequently discharged. Patient demographics are presented in Table 1.

**Table 1:** General information.

General data			
Parameter	Case 1	Case 2	Case 3
Age (days)	5	34	135
MRI/CT examination	MRI	CT	MRI
Abnormal renal development: Left/Right (location)	Right	Right	Left
Uterine malformation (yes/no)	Yes	Yes	Yes
Vaginal diaphragm (present/absent)	Present	Present	Present
Classification of Oblique Vaginal Septum	I	I	II
Extent of vaginal fluid accumulation (mm)	12×8	15x6	76x45x48
Vaginal orifice mass size (mm)	12×10×10	10×10×10	—
Urinary tract infection (Present/Absent)	Present	Present	Present
Urinary white blood cells (cells/high-power field)	116	56	—
Urine culture bacteria	—	Escherichia coli	Enterococcus faecalis
Serum Alpha-fetoprotein (ng/ml)	—	2013.00	51087.00
Surgical Approach	Direct Visual Incision and Fenestration of Oblique Septum	Direct Visual Incision and Fenestration of Oblique Septum	Cystovaginoscopy +Laparoscope-assisted Incision and Fenestration of Oblique Septum
Operative Time (minutes)	25	30	105
Hospital Stay (days)	5	6	12

## 3 Methods

### 3.1 Anesthesia method

All three patients underwent general anesthesia using endotracheal intubation combined with intravenous anesthesia. Following anesthesia induction, each patient was placed in the lithotomy position.

### 3.2 Surgical procedures

**Figure 4:**
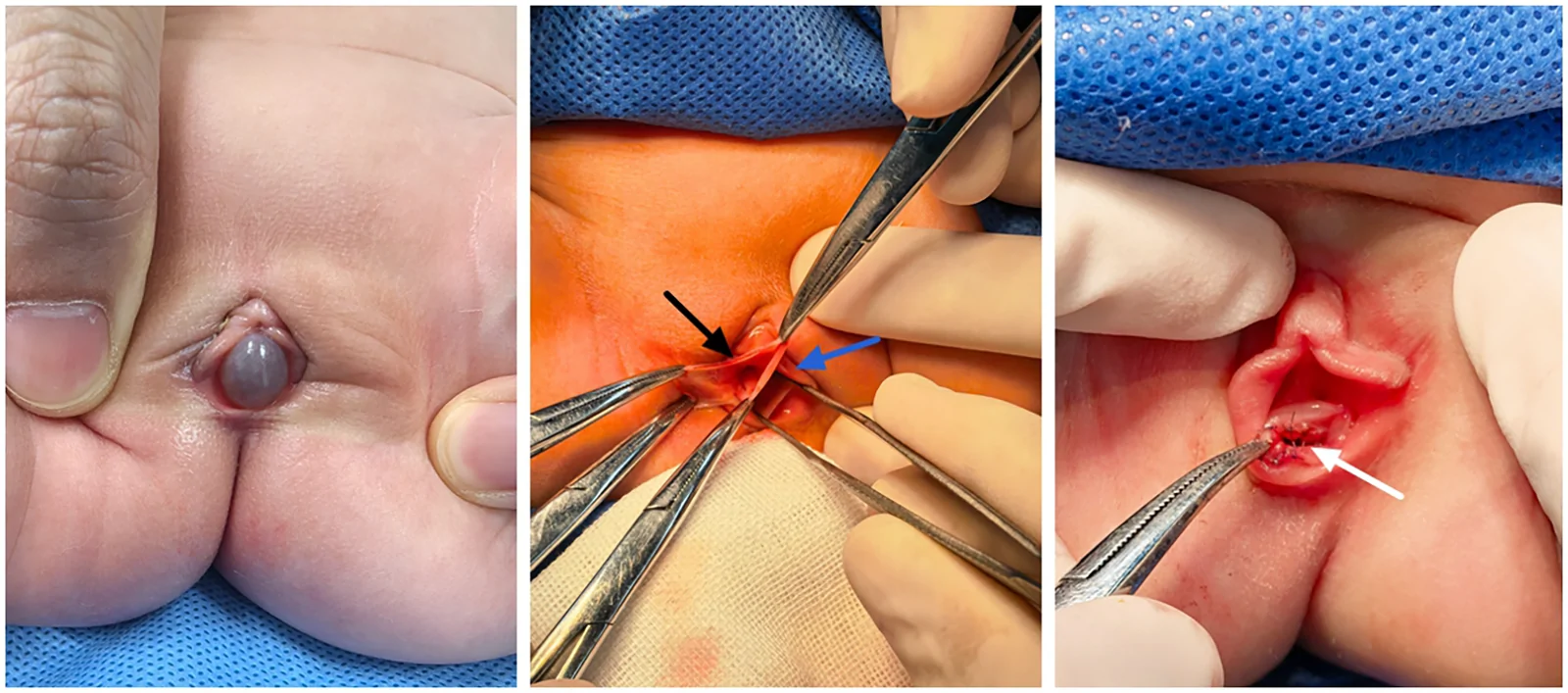
The vulvar mass is caused by protrusion of fluid accumulation in the vaginal septum **(A)**. After incision of the vaginal septum, the resulting cavity (black arrow) and the normal vaginal opening (blue arrow) are visible **(B)**. The fistula site created by vaginal septum incision (white arrow) is shown **(C)**.

**Figure 5:**
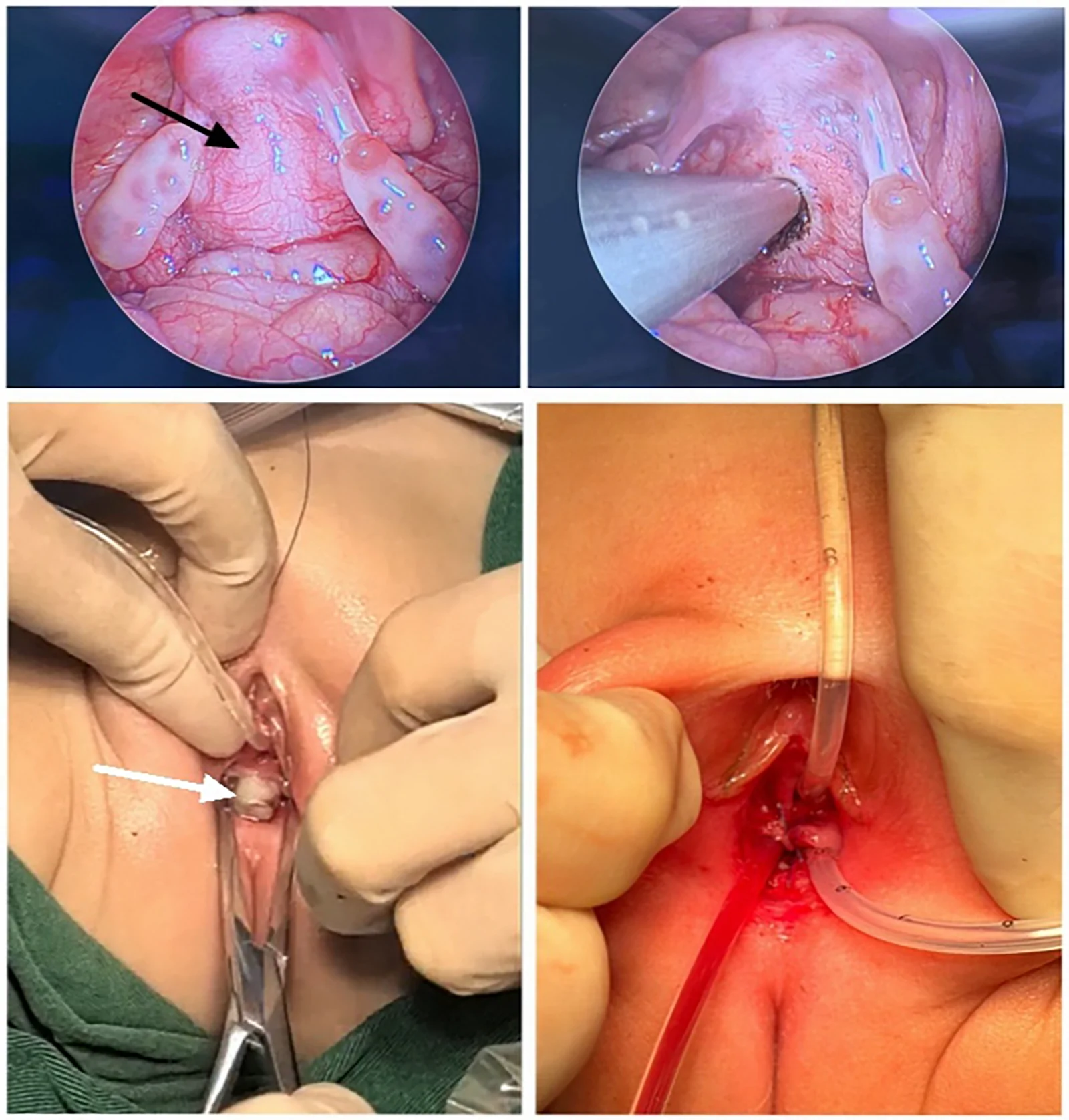
Under laparoscopy, a distended vagina is observed (black arrow) **(A)**. The posterior vaginal wall is incised, and a guidewire is inserted **(B)**. The guidewire is visible at the vulvar orifice (white arrow) **(C)**. Drain tubes are placed separately in the urethra, the vaginal oblique septum, and the normal vaginal cavity **(D)**.

1. Direct-Vision Oblique Septum Incision and Stomatoplasty (Figure 4): Cyst wall incision revealed a cavity-like structure consistent with a duplicated vagina. The protruding portion of the oblique septum was excised, and the incision margins were everted and sutured to create a stoma. The cystic cavity was packed with Vaseline gauze, and a urinary catheter was placed. The Vaseline gauze and urinary catheter were removed after three days.
2. Cystovaginoscopy Combined with Laparoscopic-Assisted Oblique Septum Incision and Stomatoplasty (Figure 5): Cystovaginoscopy was performed first: Cystoscopy revealed compression of the bladder by vaginal fluid accumulation, precluding visualization of the bilateral ureteral orifices. Vaginoscopic examination showed a narrowed vaginal canal due to compression by fluid within the oblique septum. A urinary catheter was placed. The procedure was then converted to laparoscopy: A single-port trocar was inserted at the umbilicus, and a pneumoperitoneum was established at a pressure of 7 mmHg. The uterine fundus exhibited a saddle-shaped depression centrally; bilateral ovaries appeared well-developed. A bulge was observed at the vaginal vault. A 1.0 cm incision was made at the posterior vaginal wall bulge, draining copious purulent fluid. After suctioning the pus, a guide rod was directed toward the vaginal introitus. The procedure was then converted to a transvaginal approach: The guide rod was visualized protruding through the vaginal introitus. The oblique septum was incised along the guide rod. The incision margins were everted and sutured using a locking stitch to create a stoma, and a drainage tube was placed laparoscopically to close the original vaginal incision, and a pelvic drain was inserted. The drainage tube was removed five days later.

## 4 Results

Three pediatric patients successfully underwent the surgical procedure, with operative times ranging from 25 to 105 min (mean: 53.3 min). No postoperative complications, such as hematuria or hematochezia, were observed. The mean postoperative hospital stay was 7.7 days. The follow-up period ranged from 1 to 12 months. After discharge, Patients 1 and 2 exhibited normal urination without bifurcation of the urinary stream. Physical examination revealed no tumor recurrence at the external genital opening, and routine urinalysis and urine cultures were negative. Transvaginal ultrasound showed no significant fluid accumulation. Patient 3 had no fever after discharge and maintained normal urination and defecation. At the 1-month follow-up, physical examination revealed no abdominal masses, and the external genital opening showed no redness, swelling, or purulent discharge. Routine urinalysis and urine cultures were negative. Transvaginal ultrasound revealed a small amount of fluid accumulation, measuring 22 mm × 7 mm × 14 mm. Urological ultrasound indicated that hydroureteronephrosis on the affected side was reduced compared to the preoperative status.

## 5 Discussion

Infantile Herlyn-Werner-Wunderlich syndrome, a rare congenital Müllerian duct anomaly characterized by didelphys uterus, unilateral obstructed hemivagina, and ipsilateral renal agenesis (7), is frequently underdiagnosed before puberty due to its insidious clinical presentation. Without timely intervention, the accumulation of blood or fluid resulting from obstruction can lead to recurrent urinary tract infections, dysuria, and even irreversible hydronephrosis and renal failure (8). Although magnetic resonance imaging is considered the diagnostic gold standard, the narrow anatomical structures in infancy render transvaginal procedures highly challenging (9). Traditional puncture and drainage, while providing temporary symptomatic relief, are associated with a high recurrence rate and an increased risk of exacerbating local infection. Consequently, exploring safe and effective minimally invasive surgical strategies is crucial for improving long-term outcomes in these pediatric patients (10).

This case report presents the clinical data of three infants diagnosed with oblique vaginal septum syndrome. The results demonstrate that direct vaginal incision effectively relieves obstruction in cases involving a visible lower-segment septum. However, for upper-segment septa or those complicated by complex pelvic fluid collections, laparoscopic-assisted techniques can overcome visual field limitations, enabling precise dissection and avoidance of injury to adjacent organs (11). All three infants experienced significant symptomatic relief postoperatively, and no major complications were observed during the follow-up period.

Previous studies on this condition have primarily focused on adolescent and adult patients, thereby untangling its distinctive onset patterns (12, 13). Large-scale systematic reviews have indicated that the most common initial symptoms in patients with oblique vaginal septum syndrome are dysmenorrhea and abnormal uterine bleeding, which typically manifest after menarche (12). However, in the present case report, the three infants presented with non-specific symptoms, including dysuria, external genital masses, or abdominal masses, which markedly differ from the typical symptom manifestation pattern dependent on the menstrual cycle. The core mechanism underlying this discrepancy is that, during infancy, the obstruction is not driven by the accumulation of menstrual blood. Instead, it results from the pathological accumulation of vaginal secretions or urine (in the presence of a fistula) within a closed cavity, leading to hydrocolpos or pyocolpos (14). The short anatomical length of the infantile vagina accentuates the mass effect of fluid accumulation, which readily compresses the urethra and bladder neck. This anatomical predisposition explains why dysuria is more prominent in infants, in contrast to the cyclical pelvic pain commonly observed during adolescence (5). Furthermore, in Case 3 of the present report, which presented with an abdominal mass, the obstruction was located at a higher level. This finding aligns with the MRI-based classification system, which correlates a higher obstruction site with more complex clinical presentations, thereby underscoring that the anatomical level of the obstruction is a critical determinant of symptom type and severity (14). It is our contention that in infants presenting with ipsilateral renal agenesis, urinary tract infection and dysuria should be regarded as important warning signs of Herlyn-Werner-Wunderlich syndrome, even in the absence of a menstrual history.

This report delineates the distinction between transvaginal direct incision and laparoscopy-assisted incision based on septal location and local anatomical conditions. This strategy aligns with and further refines the current consensus that vaginal septectomy is the gold-standard procedure (8, 12). The literature has established that, for the majority of patients, simple transvaginal septectomy is sufficient to relieve obstruction and alleviate symptoms (8). However, these studies are predominantly based on adult and adolescent populations, in whom the vagina is fully developed and there is minimal history of prior surgical or procedural interventions. In Case 3 of this report, 60–80 mL of fluid was aspirated monthly via puncture prior to surgery. This finding was highly suspected to be associated with an ipsilateral dysplastic kidney and an ectopic ureter opening into the vaginal septum cavity. Due to the bulging effect of the accumulated fluid compressing the vagina, conventional transvaginal surgery faced significant challenges, including severely inadequate visual field exposure and a high risk of collateral injury. To address this difficulty, we employed a laparoscopy-assisted technique, wherein a guide rod was inserted into the septum cavity to elevate the septum and facilitate transvaginal incision. In this report, the three infants exhibited complete resolution of postoperative obstructive symptoms and infection, with no recurrence during the follow-up period. This outcome confirms that adequate incision of the septum to achieve unobstructed drainage is the core therapeutic objective (13). Additionally, the literature indicates that intermediate and high vaginal obstructions carry a relatively higher risk of postoperative stenosis or retraction (14). This suggests that for patients undergoing high-level incision, short-term moderate dilation may be considered as a preventive measure; however, the potential irritation to the delicate infantile tissues must be carefully weighed. The standardization of such a strategy remains to be further explored.

An in-depth analysis of Case 3 in this report was conducted to elucidate the causes of the poor outcomes following conservative puncture and the subsequent surgical complications. The analysis identified delayed diagnosis and inappropriate invasive interventions as risk factors contributing to the clinical complexity of this case. In Case 3, the fluid collection was initially attributed to unresolved maternal estrogen in the infant, without consideration of urine from an ectopic ureter. The puncture procedure not only failed to relieve the obstruction but may have introduced infection into the previously sterile fluid collection cavity. This likely led to the progression from simple hydrocolpos to pyocolpos, accompanied by the induction of severe local inflammation and subsequent adhesions. Consequently, the following insights were gained: 1) When a diagnostic puncture yields a substantial volume of fluid, the possibility that it is urine should be excluded. If a subsequent puncture again yields a large amount of fluid, a high suspicion should be maintained for a dysplastic kidney with an ectopic ureteral orifice on the affected side, and repeated puncture and aspiration should be avoided to prevent infection. 2) In this pediatric patient, the clinical presentation suggested fluid accumulation within the oblique vaginal septum due to a dysplastic kidney with an ectopic ureteral orifice. Although a septostomy and marsupialization have been performed, postoperative urinary incontinence resulting from the ectopic ureteral orifice remains a potential concern. The substantial volume of drainage fluid observed after the septostomy indicates that the affected kidney retains partial function; therefore, further surgical management of the affected kidney is warranted.

The principal limitations of this report are the extremely limited sample size and the absence of a parallel control group subjected to traditional puncture drainage or alternative surgical approaches (e.g., vaginoscopy). Moreover, the surgical procedures performed on the three pediatric patients were confined to relieving the obstruction caused by vaginal fluid accumulation during early childhood; the vaginal septum was not completely excised. Consequently, these patients will require subsequent gynecological surgery for septal resection during adolescence or adulthood. This eventuality was thoroughly communicated to the patients' parents, and their informed consent was obtained. Furthermore, with a mean follow-up period of only 12 months, it is not feasible to evaluate the potential effects of residual or re-stenosed oblique vaginal septum after puberty on menstrual drainage and long-term fertility. Accordingly, these patients will continue to be followed prospectively.

## 6 Conclusion

This report presents two minimally invasive surgical strategies for managing oblique vaginal septum syndrome in three infants: transvaginal incision under direct visualization is suitable for patients with a low-lying oblique septum presenting as a vulvar mass, whereas a laparoscopic-assisted approach effectively optimizes surgical conditions for cases involving a high-lying oblique septum or significant inflammation following repeated puncture. Both approaches achieved symptom relief without major complications. Based on these three cases, we emphasize the importance of early and precise intervention, while avoiding blind puncture to preserve renal function. Future studies will involve larger sample sizes and extend follow-up into the post-pubertal period to further validate the safety and efficacy of these surgical strategies.

## Data Availability

The datasets presented in this article are not readily available because of ethical and privacy restrictions. Requests to access the datasets should be directed to the corresponding author.
